# A novel pathway to detect muscle-invasive bladder cancer based on integrated clinical features and VI-RADS score on MRI: results of a prospective multicenter study

**DOI:** 10.1007/s11547-022-01513-5

**Published:** 2022-06-28

**Authors:** Marco Bicchetti, Giuseppe Simone, Gianluca Giannarini, Rossano Girometti, Alberto Briganti, Eugenio Brunocilla, Gianpiero Cardone, Francesco De Cobelli, Caterina Gaudiano, Francesco Del Giudice, Simone Flammia, Costantino Leonardo, Martina Pecoraro, Riccardo Schiavina, Carlo Catalano, Valeria Panebianco

**Affiliations:** 1 Department of Radiological Sciences, Oncology and Pathology, Sapienza University/Policlinico Umberto I, Viale Regina Elena 324, 00161 Rome, Italy; 2 Department of Urology, ‘Regina Elena’ National Cancer Institute, IRCCS, Rome, Italy; 3 Unit of Urology, Santa Maria della Misericordia Academic Medical Center, Udine, Italy; 4 Institute of Radiology, Santa Maria della Misericordia Academic Medical Center, Udine, Italy; 5 Department of Urology and Division of Experimental Oncology, Urological Research Institute, IRCCS Vita-Salute San Raffaele University, Milan, Italy; 6 Department of Urology, University of Bologna, Bologna, Italy; 7 Department of Radiology, IRCCS Ospedale San Raffaele Di Turro, Milan, Italy; 8 Department of Radiology, IRCSS Vita-Salute San Raffaele University, Milan, Italy; 9 Department of Radiology, University of Bologna, Bologna, Italy; 10 Department of Maternal-Infant and Urological Sciences, Sapienza University/Policlinico Umberto I, Rome, Italy

**Keywords:** Magnetic resonance imaging, VI-RADS, Muscle-invasive bladder cancer, Hematuria, Pathway

## Abstract

**Purpose:**

To determine the clinical, pathological, and radiological features, including the Vesical Imaging-Reporting and Data System (VI-RADS) score, independently correlating with muscle-invasive bladder cancer (BCa), in a multicentric national setting.

**Method and Materials:**

Patients with BCa suspicion were offered magnetic resonance imaging (MRI) before trans-urethral resection of bladder tumor (TURBT). According to VI-RADS, a cutoff of ≥ 3 or ≥ 4 was assumed to define muscle-invasive bladder cancer (MIBC). Trans-urethral resection of the tumor (TURBT) and/or cystectomy reports were compared with preoperative VI-RADS scores to assess accuracy of MRI for discriminating between non-muscle-invasive versus MIBC. Performance was assessed by ROC curve analysis. Two univariable and multivariable logistic regression models were implemented including clinical, pathological, radiological data, and VI-RADS categories to determine the variables with an independent effect on MIBC.

**Results:**

A final cohort of 139 patients was enrolled (median age 70 [IQR: 64, 76.5]). MRI showed sensitivity, specificity, PPV, NPV, and accuracy for MIBC diagnosis ranging from 83–93%, 80–92%, 67–81%, 93–96%, and 84–89% for the more experienced readers. The area under the curve (AUC) was 0.95 (0.91–0.99). In the multivariable logistic regression model, the VI-RADS score, using both a cutoff of 3 and 4 (*P* < .0001), hematuria (*P* = .007), tumor size (*P* = .013), and concomitant hydronephrosis (*P* = .027) were the variables correlating with a bladder cancer staged as ≥ T2. The inter-reader agreement was substantial (*k* = 0.814).

**Conclusions:**

VI-RADS assessment scoring proved to be an independent predictor of muscle-invasiveness, which might implicate a shift toward a more aggressive selection approach of patients’ at high risk of MIBC, according to a novel proposed predictive pathway.

**Supplementary Information:**

The online version contains supplementary material available at 10.1007/s11547-022-01513-5.

## Background

Bladder cancer (BCa) is the 10th most diagnosed tumor in the world, and the new 5-years prevalence estimates show that 1,720,625 people are living with bladder cancer within five years of a past diagnosis, since it is a highly recurrent tumor [1]. Interestingly, BCa-related costs are caused by the hospital care and represent 5% of total health care cancer costs [2]. This is probably linked to the fact that the diagnostic pathway for bladder cancer patients has been mostly unchanged for more than 30 years, with trans-urethral resection of bladder tumor (TURBT) as the initial diagnostic and staging tool [3]. Therefore, an adequate, effective, and personalized management of this disease, from the diagnostic-staging to the therapeutic steps of the work-up, should be envisioned to bring major beneficial implications on patients’ well-being. That is why, in the last years, the role of MRI for BCa staging has represented a hot topic in the urological community. A turning point has occurred when the Vesical Imaging-Reporting and Data System (VI-RADS) was released in 2018 to improve standardization, reliability and reproducibility of MR images acquisition and reporting, to optimally differentiate non-muscle-invasive from muscle-invasive BCa (NMIBC vs MIBC) which represents the diagnostic cornerstone of the disease [4]. Since then, the VI-RADS assessment score has been validated by different groups and up to now three meta-analysis have been published showing optimal results in terms of VI-RADS diagnostic performance and inter-reader reproducibility [5–10]. The most recent meta-analyses showed a pooled weighted mean *κ* estimate of 0.83 (95% CI 0.78–0.88) and pooled sensitivity, specificity, and area under the curve (AUC) value of 0.77–0.90 (95% CI 0.65–0.94), 0.97–0.86 (95% CI 0.71–0.99), and 0.92–0.93 (95% CI 0.89–0.95) for VI-RADS 3–4 as the cutoff value for MIBC [6, 7]. However, while the accuracy of VI-RADS has been reasonably proven, it is not clear yet whether clinical and pathological features can influence VI-RADS categorization, and in turn whether the VI-RADS can better predict the risk of MIBC alone or rather in combined models including extra-radiological factors.

With this background, the aim of our study was to determine the clinical, radiological, and pathological features associated with muscle-invasive bladder cancer. We tested the hypothesis that multi-level variables might improve the current treatment paradigm of BCa in the pre-treatment setting.

## Materials and methods

### Study design and patients population

This prospective multicenter multireader observational study received formal approval from the Institutional Review Board and the Ethical Committee of each participating center. All patients were notified of the investigational nature of this study and gave their written informed consent. The study was conducted in accordance with the guidelines for good clinical practice in accordance with ethical principles as reported in the latest version of the Declaration of Helsinki. All patients with suspicion of BCa were referred to four different centers (Sapienza University of Rome [center #1], "Regina Elena" National Cancer Institute of Rome [center #2], Santa Maria della Misericordia University Hospital in Udine [center #3] and the University Hospital of Bologna [center #4]), between January 2020 and May 2021, and were offered bladder MRI. The inclusion criteria were a primary diagnosis of bladder tumor, positive urinary cytology, suspected bladder neoplasm identified by ultrasound of the urinary tract and/or cystoscopy and/or CT scan of the abdomen-pelvis. The exclusion criteria were history of prior urinary tract neoplasms, impossibility of achieving appropriate bladder distension, concomitant diagnosis of carcinoma in situ (CIS), no detectable lesion on MRI and any contraindication to MRI (low renal function, MR unsafe medical devices etc.) and to spinal and general anesthesia. No previous study was conducted on the same population cohort.

### MR imaging acquisition protocol and image analysis

MRI of the pelvis was performed using a 3 Tesla magnet (Discovery MR750, GE Healthcare, Milwaukee, WI) (center #1 and #2), using a 3 Tesla magnet (Achieva, Philips) (center #3), both equipped with a 32-channel phased-array body coil, and a 1.5 Tesla scanner (Signa HDxt; GE Healthcare, Milwaukee, WI) equipped with an 8-channel phased-array surface coil (center #4). The MR imaging protocol was in accordance with the original VI-RADS document [4]. Patients were administered an intramuscular antispasmodic (n-butyl-scopolamine 20 mg) agent when necessary to lower bladder wall motion and were instructed to drink 500–1000 ml of water 30 min before the examination to obtain adequate bladder distension. A summary Supplementary Table 1 details the MRI acquisition parameters.

Image analysis was performed independently by two radiologists in each center, one more experienced (ME) and one less experienced (LE), respectively, with 15 and 4 (center #1 and #2), 15 and 5 (center #3) and 10 and 5 years of experience (center #4), respectively. Cumulative reading experience for less experienced readers ranged from 20 to 80 cases. MRIs of patients enrolled in center #2 were acquired and analyzed by radiologists from center #1. Each reader analyzed images blindly to clinical data, following the evaluation algorithm of the VI-RADS system. VI-RADS cutoff scores of 3 or greater and 4 or greater were used to define muscle invasiveness in our analysis. For patients with more than one lesion, only the lesion with the highest VI-RADS score was used.

### Standard of reference (TURBT, Re-TURBT and radical cystectomy)

All enrolled patients underwent primary conventional TURBT (white light) within 6 weeks after bladder MRI. All endoscopic resection procedures were performed by a single operator designated for each enrollment center. Each endoscopic resection procedure was performed adhering to the dictates of the EAU guidelines [11]. Patients who were candidates for Re-TURBT underwent secondary resection of the tumor within 2–6 weeks of TURBT. The following cases were considered candidates for Re-TURBT, according to EAU guidelines: incomplete endoscopic resection at first TURBT, T1 patients histologically established by TURBT, and patients without detrusor muscle inclusion in the histologic specimen of the first TURBT except for low-grade Ta. All Re-TURBTs were performed by the same operator who previously performed the TURBT using conventional resection technique with monopolar or bipolar current. The site of the previous resection area identified as corresponding to the lesion characterized by the highest VI-RADS score was separately sampled and analyzed. All pathology reports performed by a dedicated pathologist with more than 10 years of experience in the field (TURBT, Re-TURBT and RC) defined staging of the disease according to the TNM classification [12] and the WHO 2004 classification (grading system) to determine the degree of cellular anaplasia [13]. Standard of reference was considered the pathology report of the primary and/or secondary TURBT, or RC when performed.

### Statistical analysis

The performance of MRI was assessed by means of receiver operating characteristic curve analysis for both the more and less experienced readers. Sensitivity, specificity, positive predictive value (PPV), negative predictive value (NPV), and accuracy were calculated to assess the performance of VI-RADS scoring by each reader. In addition, a per-sequence receiver operating characteristic curve analysis was performed. Inter-reader agreement analysis between the more and less experienced readers was performed with Cohen’ K statistics for overall VI-RADS assessment and on a per-sequence basis, to investigate scoring variability. Correlation between patient demographics, clinical characteristics and VI-RADS was analyzed using Pearson’s Chi-squared test. The normality of the variables was tested. Two univariable analyses were performed: the first included clinical/pathological features (patient age and sex, history of smoking [number of cigarettes, years of smoking], Charlson Comorbidity Index [CCI], body mass index [BMI], presence of hematuria [micro- and macrohematuria], number of foci at TURBT, grading [low/high and G1/2/3]), and the overall VI-RADS categories; the second included VI-RADS and all radiological features (lesion size, the presence of tumor peduncle and inner layer, hydronephrosis, and suspicious lymph nodes). For both analyses, a multivariable logistic regression model including only significative variables at univariable analysis adjusted for age and sex was implemented to determine the features that had independent effect on MIBC detection. All statistical analyses were performed using Statistical Package for the Social Sciences (SPSS) version 28. All tests were two-sided, and statistical significance was set at *P* < 0 0.05.

## Results

### Patient characteristics

Overall, 151 consecutive patients were enrolled and underwent MRI (72 patients from center #1, 51 patients from center #2, 16 from center #3 and 12 from center #4). Out of these patients, 12 (8%) had at least one exclusion criteria and were excluded. Thus, 139 patients were included in the final patient sample. The median ages were 70 years (interquartile range, 64–76.5 years) among 103 (74%) males and 36 (26%) female patients. Patient characteristics are shown in Table 1, according to invasiveness of the disease.

**Table 1:** Patients’ demographics and baseline characteristics

	NMIBC, *n* (%)	MIBC, *n* (%)
*No. of patients*	97	42
*Age (mean)*		
< 70	43 (44.3)	17 (40.5)
≥ 70	54 (55.7)	25 (59.5)
*Sex*		
Male	70 (72)	33 (78.6)
Female	27 (28)	9 (21.4)
*Smoking history*		
No	26 (26.9)	12 (28.6)
Yes	71 (73.1)	30 (71.4)
*Number of cigarettes per day*		
< 15	31 (43.7)	6 (20)
≥ 15	40 (56.3)	24 (80)
*Years of smoking*		
< 20	21 (29.6)	5 (16.7)
≥ 20	50 (70.4)	25 (83.3)
*Charlson comorbidity index*		
< 5	59 (60.8)	19 (45.2)
≥ 5	38 (39.2)	23 (54.8)
*Body mass index (kg/m*^2^)		
< 18	3 (3.0)	1 (2.4)
18–25	43 (44.4)	19 (45.2)
> 25	51 (52.6)	22 (52.4)
*Hematuria*		
No	25 (25.8)	3 (7.1)
Micro	26 (26.8)	5 (11.9)
Macro	45 (46.4)	34 (81)
*WHO grading (2004)*		
Low grade	41 (42.2)	1 (2.4)
High grade	56 (57.8)	41 (97.6)
*WHO grading (1973)*		
G1	14 (14.4)	0 (0)
G2	23 (23.7)	3 (7.1)
G3	60 (61.9)	39 (92.9)
*No. of foci at TURBT*		
1	64 (65.9)	38 (90.5)
> 1	33 (34.1)	4 (9.5)

NMIBC, non muscle-invasive bladder cancer; MIBC, muscle-invasive bladder cancer; WHO, World Health Organization; TURBT, trans-urethral resection of bladder tumor; G, grade

### Diagnostic performance of VI-RADS assessment

**Fig. 1:**
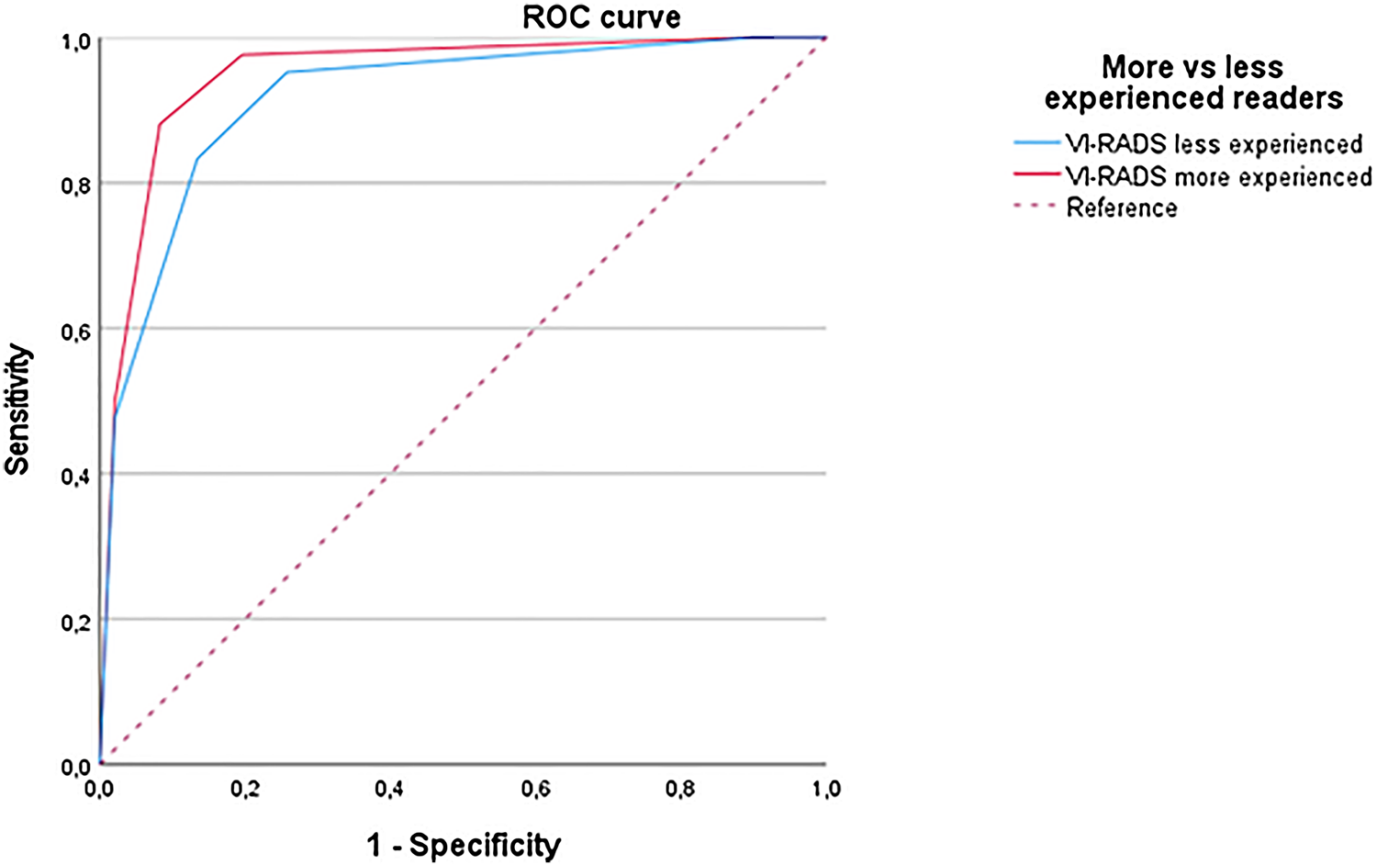
ROC analysis for the performance of more and less experienced readers in detecting muscle-invasive bladder cancer. ROC, receiver operating curve; VI-RADS, Vesical Imaging-Reporting and Data System

The performance of VI-RADS assessment in detecting muscle-invasive bladder cancer using VI-RADS 3 and 4 as cutoff for muscle invasion detection is presented in detail in Table 2. The AUCs for detecting MIBC of more and less experienced readers were 0.95 (95% CI 0.91, 0.99) and 0.93 (95% CI 0.88, 0,98), respectively. Figure 1 shows the receiver operating characteristic curves of overall VI-RADS assessment for both more and less experienced readers. On a per-sequence analysis, overall VI-RADS showed the highest AUC compared to the single sequences (T2WI: 0.94 [95% CI 0.90, 0.98]; DWI: 0.93 [95% CI 0.87, 0.98]; DCE: 0.94 [95% CI 0.90, 0.98]) for both groups of readers; details are shown in Supplementary Table 2.

**Table 2:** Diagnostic performance of VI-RADS assessment performed by the more and less experienced readers

	AUC (95% CI)	VI-RADS cutoff	SENS (%)	SPEC (%)	PPV (%)	NPV (%)	ACC (%)
Experienced readers	0.95 (0.91–0.99)						
		3	93 (39/42)	80 (78/97)	67 (39/58)	96 (78/81)	84 (117/139)
		4	83 (35/42)	92 (89/97)	81 (35/43)	93 (89/96)	89 (124/139)
Less experienced readers	0.93 (0.88–0.98)						
		3	95 (40/42)	73 (71/97)	60 (40/66)	97 (71/73)	80 (111/139)
		4	83 (35/42)	86 (83/97)	71 (35/49)	92 (83/90)	85 (118/139)

AUC, area under the curve; CI, confidence interval; VI-RADS, Vesical Imaging-Reporting and Data System; Send, sensitivity; Spec, specificity; PPV, positive predictive value; NPV, negative predictive value; ACC, accuracy

**Fig. 2:**
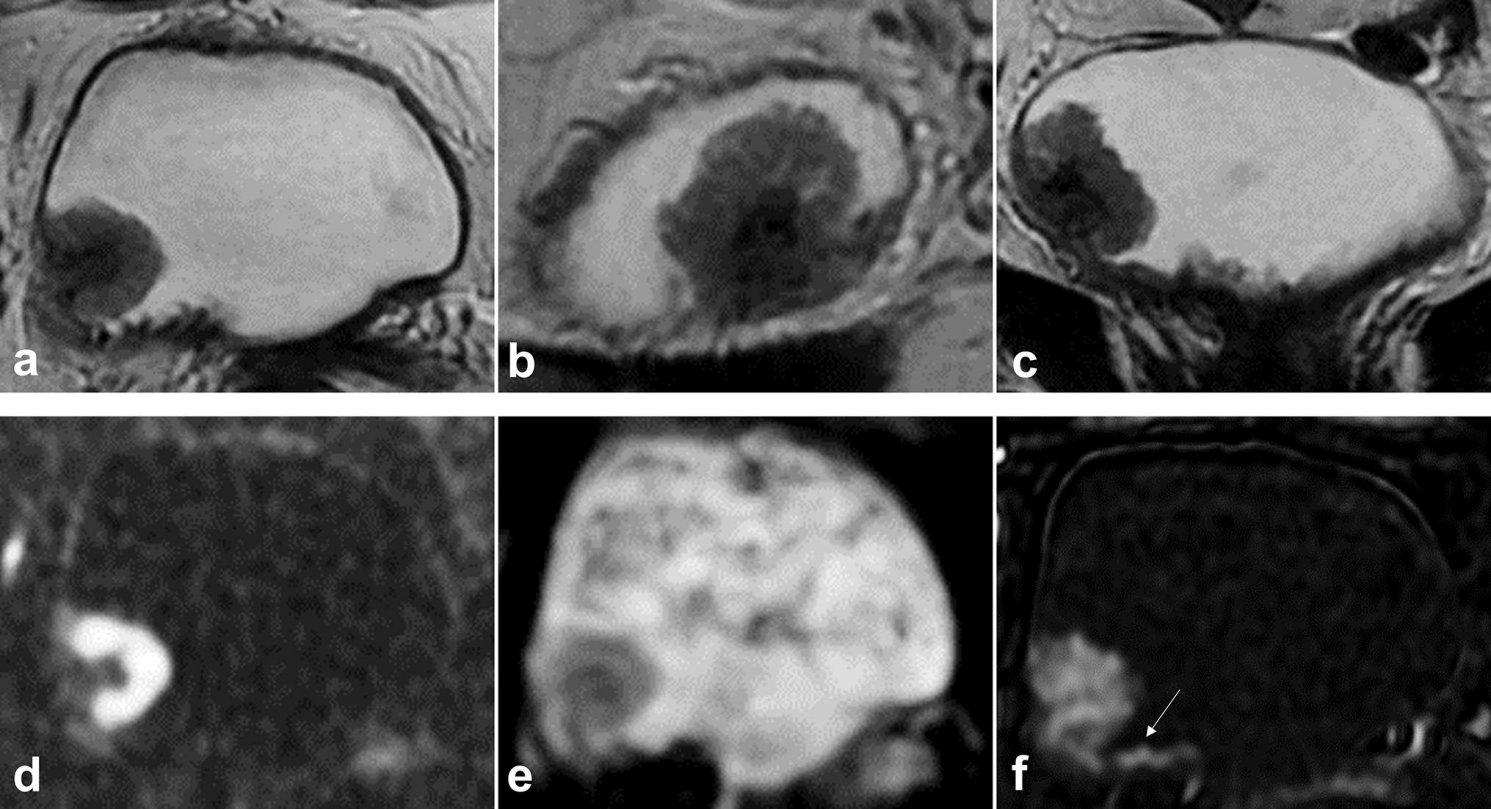
Case example of a 66-years-old female that was incorrectly scored as a VI-RADS 4 by the less experienced readers, but correctly scored as VI-RADS 2 by the more experienced readers. (**a**–**b**–**c**) Axial, sagittal and coronal T2-weighted imaging showing a pedunculated bladder tumor (20 mm) at the right lateral wall; (**d**–**e**) diffusion-weighted imaging and ADC map showing the “inchworm sign”, typical of non-muscle-invasive bladder cancer; (**f**) dynamic contrast-enhanced MRI showing integrity of the *muscularis propria* layer and enhancement of the “inner layer” (arrow). The lesion should be classified as VI-RADS 2. VI-RADS, Vesical Imaging-Reporting and Data System; ADC, apparent diffusion coefficient

Inter-reader agreement between the more and less experienced radiologists using *k* statistics was 0,814 (*P* < 0.001) for overall VI-RADS assessment. Supplementary Tables 2 and 3 summarize the *k* statistics for each MRI sequence and overall VI-RADS assessment. Figure 2 shows a case in which there was no agreement between the readers.

### Univariable and multivariable analysis for determining the clinical, radiological, and pathological features associated with muscle-invasive bladder cancer

On univariable analysis performed using clinical features, pathology data and VI-RADS, the number of cigarettes smoked per day (< 15 or ≥ 15) (*P* = 0.02), concomitant hematuria (either micro or macro) (*P* = 0.002), the number of TURBT foci (1 or > 1) (*P* = 0.002), the WHO scoring grading (low or high grade) (*P* < 0.0001), the 1973 grading (G1 vs. G2 vs. G3) (*P* = 0.001), and the VI-RADS assessment using both cutoff 3 and 4 (*P* < 0.0001) correlated with MIBC. In the multivariable logistic regression model, the variables showing independent correlation with MIBC were concomitant hematuria (odds ratio [OR]: 11.0 [95% CI 1.93–62.73]; *P* = 0.007) and the VI-RADS assessment with a cutoff of 3 or greater (OR: 55.15 [95% CI 9.17, 331.54]; *P* < 0.0001). Using a VI-RADS cutoff of 4, the only independent variable was the VI-RADS scoring itself (OR: 39.27 [95% CI 8.13, 189.80]; *P* < 0.0001).

On univariable analysis performed using only radiological features, MIBC correlated with the VI-RADS assessment using both cutoff 3 and 4 (*P* < 0.0001), with the lesion size (< 2.5 cm or ≥ 2.5 cm) (*P* < 0.0001), with the presence of the vascular peduncle (*P* < 0.0001) and the inner layer (*P* = 0.001), and with the presence of suspicious lymph nodes (*P* < 0.0001). In the multivariable logistic regression model, a VI-RADS score confirmed to be independent predictor of MIBC using both cutoff of 3 and 4 (cutoff of 3: OR: 15.98 [95% CI 3.90, 65.94]; *P* < 0.0001; cutoff of 4: OR: 22.16 [95% CI 6.12, 80.27]; *P* < 0.0001). In addition, using both cutoff, lesion size showed independent correlation (cutoff of 3: OR: 4.32 [95% CI 1.36, 13.65]; *P* = 0.013; cutoff of 4: OR: 4.43 [95% CI 1.27, 15.42]; *P* = 0.02). Instead, only when applying a VI-RADS a cutoff of 4, also the concomitant presence of hydronephrosis was a predictor of MIBC (OR: 19.07 [95% CI 1.40, 259.72]; *P* = 0.02). Age and sex, smoking history alone and total years of smoking, CCI, and BMI were not predictive of MIBC. Tables 3 and 4 show the results of both univariable and multivariable analyses.

**Table 3:** Univariable and multivariable regression analyses assessing the correlation among clinic-pathological factors and VI-RADS with muscle invasive bladder cancer. MIBC, muscle invasive bladder cancer; TURBT, trans-urethral resection of bladder tumor; WHO, World Health Organization; VI-RADS, Vesical Imaging-Reporting and Data System

Clinical, pathological factors and VI- RADS correlation with MIBC	p*	Odd ratio**	p**
Age	0.699		
Sex	0.486		
Smoking history	0.848		
Number of cigarettes per day	0.027	1.44 (0.29–6.26)^∋^ 2.76 (0.56–13-45)^σ^	0.709^∋^ 0.209^σ^
Years of smoking	0.215		
Charlson comorbidity index	0.084		
Body mass index	0.957		
Hematuria	0.002	11.0 (1.93–62.73)^∋^ 5.14 (0.92–28.58)^σ^	0.007^∋^ 0.062^σ^
No. of foci at TURBT	0.002	0.22 (0.03–1.60)^∋^ 0.22 (0.03–1.71)^σ^	0.136^∋^ 0.148^σ^
WHO grading (2004)	< 0.0001	2.35 (0.16–33.62)^∋^ 3.37 (0.25–45.02)^σ^	0.528^∋^ 0.358^σ^
WHO grading (1973)	0.001	6.99 (0.86–56.35)^∋^ 4.95 (0.58–41.72)^σ^	0.068^∋^ 0.141^σ^
VI-RADS cutoff 3†	< 0.0001	55.15 (9.17–331.54)	< 0.0001
VI-RADS cutoff 4†	< 0.0001	39.27(8.13–189.80)	< 0.0001

**†**Experienced readers *Univariable analysis **Multivariable analysis ^∋^VI-RADS cutoff 3 ^σ^VI-RADS cutoff 4

**Table 4:** Univariable and multivariable regression analyses assessing the correlation among radiological factors, including VI-RADS assessment score, with muscle invasive bladder cancer

VI-RADS and additional radiological factor correlation to MIBC	Muscle invasiveness status		p*	Odd ratio**	p**
	NMIBC, *n* (%)	MIBC, *n* (%)			
*No. of patients*	97	42			
*VI-RADS 1†*	10 (10.3)	0			
*VI-RADS 2†*	68 (70.1)	1 (2.4)			
*VI-RADS 3†*	11 (11.3)	4 (9.5)	< 0.0001	15.98 (3.90–65.94)	< 0.0001
*VI-RADS 4†*	6 (6.2)	16 (38.1)	< 0.0001	22.16 (6.12–80.27)	< 0.0001
*VI-RADS 5†*	2 (2.1)	21 (50)			
*Tumor size*	–	–	< 0.0001		
< 2.5 cm	61 (62.9)	10 (23.8)		4.32 (1.36–13.65)^∋^	0.013^∋^
≥ 2.5 cm	36 (37.1)	32 (76.2)		4.43 (1.27–15.42)^σ^	0.020^σ^
*Tumor stalk*			< 0.0001		
No	37 (38.1)	35 (83.3)		0.26 (0.06–1.03)^∋^	0.056^∋^
Yes	60 (61.9)	7 (16.7)		0.32 (0.07–1.39)^σ^	0.129^σ^
*Inner layer*			0.001		
No	44 (45.4)	31 (73.8)		0.67 (0.18–2.45)^∋^	0.546^∋^
Yes	53 (54.6)	11 (26.2)		0.75 (0.18–3.08)^σl^	0.691^σ^
*Hydronephrosis*			0.001		
No	96 (99.0)	35 (83.3)		8.82 (0.70–111.49)^∋^	0.092^∋^
Yes	1 (1.0)	7 (16.7)		19.07 (1.40–259.72)^σ^	0.027^σ^
*Suspicious LNs*			< 0.0001		
No	92 (94.8)	31 (73.8)		2.31 (0.53–10.01)^∋^	0.263^∋^
Yes	5 (5.2)	11 (26.2)		2.23 (0.41–12.19)^σ^	0.354^σ^

NMIBC, non-muscle-invasive bladder cancer; MIBC, muscle invasive bladder cancer; TURBT, trans-urethral resection of bladder tumor; VI-RADS, Vesical Imaging-Reporting and Data System; LN, lymph nodes

†Experienced readers *Univariable analysis **Multivariable analysis ^∋^VI-RADS cutoff 3 ^σ^VI-RADS cutoff 4

## Discussion

In recent years, bladder MRI and the VI-RADS assessment score have gained interests in the scientific community since they represent the imaging tools that provide the best diagnostic performance for bladder cancer local staging, accurately differentiating NMIBC from MIBC.

**Fig. 3:**
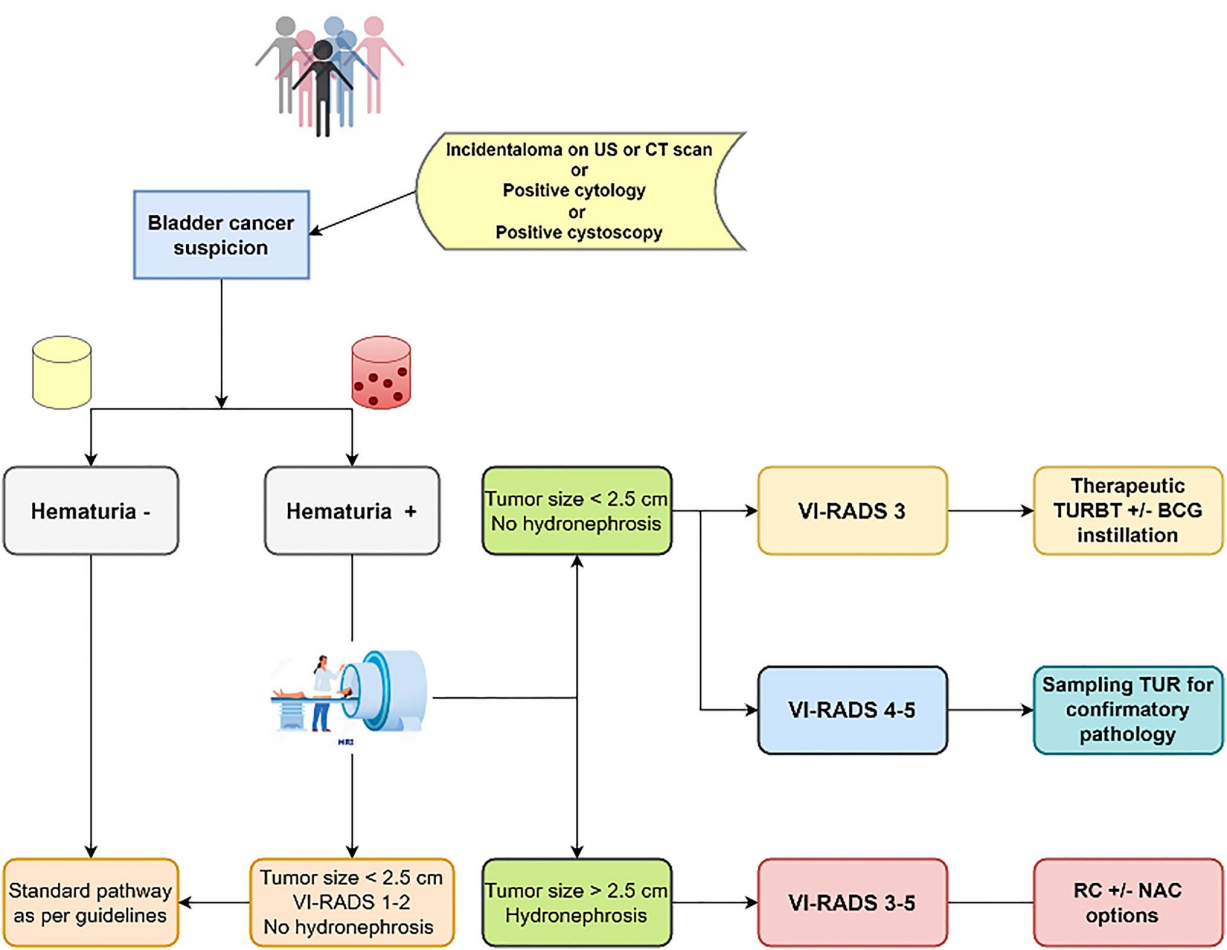
Proposed predictive pathway based on the clinical and radiological variable showing independent correlation to MIBC. US, ultrasound; CT, computerized tomography; VI-RADS, Vesical Imaging-Reporting and Data System; TURBT, trans-urethral resection of bladder tumor; BCG, bacillus Calmette-Guérin; TUR, trans-urethral resection; RC, radical cystectomy; NAC, neoadjuvant chemotherapy

In our analysis, the VI-RADS score confirmed the high diagnostic performance already demonstrated in many other investigations [14–20]. Indeed, we found that assessing bladder MRI using the VI-RADS score provided a sensitivity ranging 83–93%, specificity 80–92%, PPV 67–81%, NPV 93–96%, and accuracy of 84–89% for the more experienced readers; the less experienced readers showed a sensitivity ranging 83–95%, specificity 73–86%, PPV 60–71%, NPV 92–97%, and accuracy 80–85%. The AUC was 0.95 (0.91–0.99) for the more experienced readers and 0.93 (0.88–0.98) for the less experienced readers. The inter-reader agreement was substantial (*k* = 0.814). These results are in line with what found in the other two multicenter multireader studies. Recently, Metwally et al. prospectively enrolled 331 patients and showed that the VI-RADS score is reliable (*k* = 0.93) among four readers, and valid (sensitivity of 84%, and specificity of 92%) when using a cutoff of 3 for defining muscularis propria invasion [21]. Also, Ueno et al. showed a moderate to substantial agreement (k = 0.55–0.75) between seven radiologists (experienced and inexperienced readers), with a pooled AUC of 0.88 (0.82, 0.91) and 0.84 (0.83, 0.85) for experienced and inexperienced readers, respectively [22]. However, the primary aim of this study was to provide evidence on this topic beyond validating the score itself. Indeed, we pursued to identify, among clinical, pathological, and radiological variables, the factor independently correlating with muscle-invasive bladder cancer. The logistic regression models showed how VI-RADS score, using both a cutoff of 3 and 4 (*P* < 0.0001), hematuria (*P* = 0.007), tumor size (*P* = 0.013), and concomitant hydronephrosis (*P* = 0.027) were the variables correlating with a bladder cancer staged as ≥ T2 at either TURBT, re-TURBT, or radical cystectomy. In the past several decades, many different prognostic tools have been proposed in diverse forms, such as risk grouping/stratification [23, 24], artificial neural networks [25, 26], neurofuzzy models [27, 28], and probability nomogram [29, 30]. Often, such models were developed to predict either NMIBC recurrence, response to therapy, or overall survival [31]. However, there are no tools nowadays that efficaciously incorporate clinic-pathological data to MRI biomarkers such as the VI-RADS score, tumor size, and the presence of hydronephrosis, to preoperatively predict MIBC and to envision a tailored diagnostic pathway in which patients presenting with such characteristics would be directed to sampling TUR for confirmatory pathology, avoiding the complications related to deep TURBT, and finally to identify high-risk patients who should be directed to early radical treatment. In this regards, VI-RADS score 5 has been previously correlated with a significant delay in time to cystectomy (OR 2.81, 95% CI 1.20, 6.62). [32]. This approach might overcome the issues encountered in the first and sole clinical trial assessing whether TURBT can be replaced by MRI; indeed, preliminary results from the BladderPath trial suggested that MRI causes high rate of false-positivity; however, definitive conclusions can be made only once the enrollment of the final sample size will be done [33]. The proposed pathway might facilitate the shared decision process, potentially improving clinical outcomes of patients affected with BCa (depicted in detail in Fig. 3).

Our analyses are based on data from four institutions, even though MRIs were acquired and interpreted in three centers, without a homogeneous patients’ enrollment, thus limiting the immediate clinical application of our findings. In addition, MRI does not possess the sufficient spatial resolution to diagnose carcinoma in situ, which are per-definition high-risk cases. Also, no conclusion can be drawn on long-term prognosis since no analysis was performed on patients’ overall survival, nor recurrence rates due to limited follow-up data. Finally, the proposed predictive pathway lacks internal and external validation, and assessment of its clinical utility is warranted before it can be incorporated into routine clinical practice. Further research will focus on the independent validation of the findings.

## Conclusion

The VI-RADS assessment score was confirmed to be an accurate preoperative tool in predicting bladder cancer invasiveness, in a multicentric setting where MRI acquisition- and reporting-related biases can be overcome. Also, if considered as an MRI biomarker, and associated with clinical data and additional imaging features, VI-RADS can be incorporated into a predictive diagnostic pathway to perform a tailored patients’ selection to therapy, with the aim of preventing disease understaging and improving clinical outcomes of patients affected with bladder cancer.

## Supplementary Information

Below is the link to the electronic supplementary material.

Supplementary file1 (DOCX 1323 KB)
