# Supplementary material for: A novel pathway to detect muscle-invasive bladder cancer based on integrated clinical features and VI-RADS score on MRI: results of a prospective multicenter study
Source: Radiol Med. 2022 Jun 28;127(8):881–90. doi: 10.1007/s11547-022-01513-5 (PMC9349064; doi:10.1007/s11547-022-01513-5)

**Supplementary Material**

| **MRI Sequence** | **Acquisition Parameters** | |
| --- | --- | --- |
| **T2WI (TSE)*** | TR (ms) | 4690-5000 |
|  | TE (ms) | 80-120 |
|  | Section thickness (mm) | 3-4 (0-0.4 gap) |
|  | FOV (cm) | 23 |
|  | Matrix | 256 x 189-256 |
| **DWI (EPI)** | TR (ms) | >3000 |
|  | TE (ms) | 60-90 |
|  | Section thickness (mm) | 3-4 (0-0.4 gap) |
|  | FOV (cm) | 27-32 |
|  | Matrix | 128 x 109-128 |
|  | low b-value (sec/mm2) | 50 |
|  | Intermediate b-value (sec/mm2) | 500-800 |
|  | high b-value (sec/mm2) | 1000 |
| **DCE (GRE T1WI)** | TR (ms) | <4 |
|  | TE (ms) | 1.2 |
|  | Section thickness (mm) | 1-2, no gap |
|  | FOV (cm) | 27-35 |
|  | Matrix | 192-256 x 192-214 |
|  | Temporal resolution (s) | 5-9 |
|  | Dose (mmol/kg) | 0.1 |
|  | Injection rate (ml/s) | 3 |

* T2WI are acquired on axial, coronal, and sagittal planes

**Supplementary Table 1**. MRI protocol acquisition parameters.

| **Sequences** | **AUC (95% CI)** | | **Cohen’s k (*p*)** |
| --- | --- | --- | --- |
|  | **More experienced readers** | **Less experienced readers** |  |
| T2WI | 0.94 (0.90-0.98) | 0.92 (0.87-0.96) | 0.779 (<.001) |
| DWI | 0.93 (0.87-0.98) | 0.88 (0.82-0.95) | 0.841 (<.001) |
| DCE | 0.94 (0.90-0.98) | 0.92 (0.88-0.97) | 0.809 (<.001) |
| Overall VI-RADS | 0.95 (0.91-0.99) | 0.93 (0.88-0.98) | 0.814 (<.001) |

**Supplementary Table 2.** AUC values for each MRI sequence and overall VI-RADS scoring, for readers with different years of experience, and relative k statistic. AUC, Area Under the Curve; CI, Confidence Interval; T2WI, T2-weighted imaging; DWI, diffusion-weighted imaging; DCE, dynamic contrast-enhanced; VI-RADS, vesical imaging-reporting and data system

|  |  | **Less experienced readers, *n*** | | | | |  | **Agreement** |
| --- | --- | --- | --- | --- | --- | --- | --- | --- |
|  |  | VI-RADS 1 | VI-RADS 2 | VI-RADS 3 | VI-RADS 4 | VI-RADS 5 | Total | Cohen’s k (*p*) |
| **More experienced readers** | VI-RADS 1 | 10 | 0 | 0 | 0 | 0 | 10 | 0.814 (<.001) |
|  | VI-RADS 2 | 0 | 61 | 5 | 3 | 0 | 69 |  |
|  | VI-RADS 3 | 0 | 1 | 10 | 4 | 0 | 15 |  |
|  | VI-RADS 4 | 0 | 2 | 2 | 18 | 0 | 22 |  |
|  | VI-RADS 5 | 0 | 0 | 0 | 1 | 22 | 23 |  |
|  | Total | 10 | 64 | 17 | 26 | 22 | 139 |  |

**Supplementary Table 3:** Summary table of the VI-RADS assessment scoring between more and less experienced readers with Cohen’s k statistics. VI-RADS, vesical imaging-reporting and data system


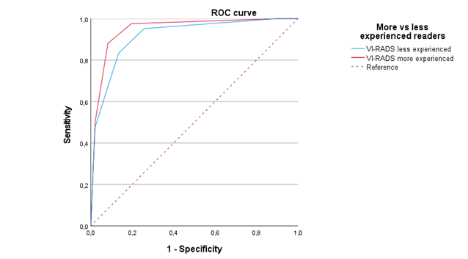


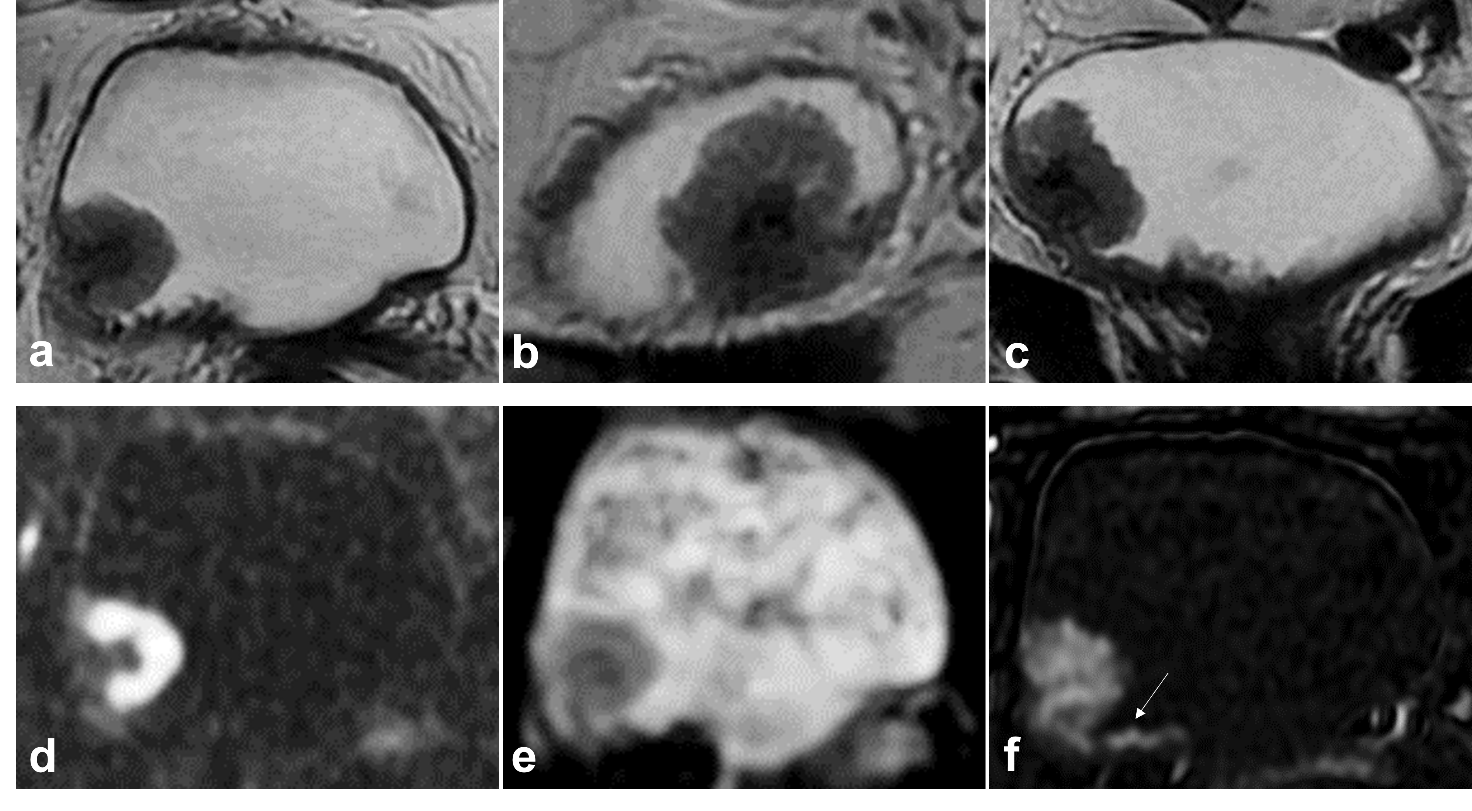


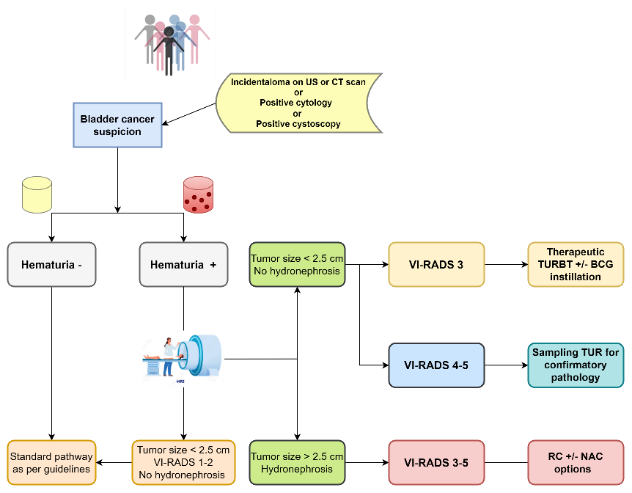

Supplement: Supplementary file 1 — Supplementary file1 (DOCX 1323 KB) [file 11547_2022_1513_MOESM1_ESM.docx]
